# Infant Mortality and Natural Selection

**Published:** 1915-12

**Authors:** 


### The Department of Eugenics, Mental and Social Hygiene

CONDUCTED BY
DR. MALONE DUGGAN
813-814 Gibbs Building, San Antonio, Texas
All communications for this department should be sent to DR. DUGGAN

### Infant Mortality and Natural Selection.

To state that two-thirds of all deaths are due to heredity is rather startling. The casual observer would not likely attribute many deaths to this cause, but rather to conditions of environment.

Investigators, especially the school of Kare Pearson, have shown this to be true, and it is explained by them by the operation of natural selection in man. Death rate is selective, and physical fitness is the criterion for survival. A heavy mortality leaves behind it a stronger population. The evidence lies in the fact that a high death rate during infancy is followed by a lower death rate during childhood. Part of children born in any district in a given year are doomed by heredity to premature death. This is due to a congenital lack of resistance to infectious diseases. In families of royalty, where environment is presumably the best, the death rate from heredity causes is 60 per cent.

This is not taken to mean that a high infant mortality is favored as a means of improving the race, but to show how the law of natural selection operates in man just the same as in the lower animal and vegetable kingdoms.

